# Health-related quality of life in surgically treated asymptomatic meningioma patients: A population-based matched cohort study

**DOI:** 10.1093/nop/npae047

**Published:** 2024-11-15

**Authors:** Olivia Näslund, Stina Jakobsson, Erik Thurin, Thomas Skoglund, Jenny Pettersson-Segerlind, Boel Brynedal, Asgeir S Jakola, Jiri Bartek

**Affiliations:** Institute of Neuroscience and Physiology, University of Gothenburg, Gothenburg, Sweden; Department of Surgery, Östra/Sahlgrenska University Hospital, Gothenburg, Sweden; Department of Plastic and Reconstructive Surgery, Karolinska University Hospital, Stockholm, Sweden; Department of Molecular Medicine and Surgery, Karolinska Institute, Stockholm, Sweden; Institute of Neuroscience and Physiology, University of Gothenburg, Gothenburg, Sweden; Department of Radiology, Sahlgrenska University Hospital, Gothenburg, Sweden; Institute of Neuroscience and Physiology, University of Gothenburg, Gothenburg, Sweden; Department of Neurosurgery, Sahlgrenska University Hospital, Gothenburg, Sweden; Department of Clinical Neuroscience and Medicine, Karolinska Institute, Stockholm, Sweden; Department of Neurosurgery, Karolinska University Hospital, Stockholm, Sweden; Department of Global Public Health, Karolinska Institute, Sweden; Institute of Neuroscience and Physiology, University of Gothenburg, Gothenburg, Sweden; Department of Neurosurgery, Sahlgrenska University Hospital, Gothenburg, Sweden; Department of Clinical Neuroscience and Medicine, Karolinska Institute, Stockholm, Sweden; Department of Neurosurgery, Karolinska University Hospital, Stockholm, Sweden; Department of Neurosurgery, Copenhagen University Hospital, Copenhagen, Denmark

**Keywords:** asymptomatic, health-related quality of life, incidental, meningioma, population-based

## Abstract

**Background:**

Asymptomatic patients with meningiomas are increasingly detected, where management can be challenging in terms of surgery versus watchful waiting. Health-related quality of life (HRQoL) is an important factor in clinical decision-making, albeit not greatly studied in this patient group. The aim of this paper is to map the HRQoL among patients with surgically removed asymptomatic meningioma as compared to the general population.

**Methods:**

Patients with first-time surgically treated asymptomatic meningioma between 2007 and 2013 were identified. Patients were invited in 2017 to answer a survey regarding different aspects of quality of life, using EuroQoL (EQ)-5D-3L, perceived health, lifestyle, and occupancy. Data from electronic patient records was obtained. The patients were matched based on age and gender with data from the Stockholm Region Public Health Cohort database.

**Results:**

There was no difference in EQ-5D-3L or visual analog scale between the patients and their matched controls. Patients and controls experienced ill health to the same extent, but patients felt to a greater extent that this impacted their way of life. In 36% of patients, preoperative occupation was not resumed, mostly due to cognitive symptoms. Additionally, the study suggested social detachment in this cohort, as significantly more patients were living alone and had less emotional support compared to controls.

**Conclusions:**

Although surgically treated patients with asymptomatic intracranial meningioma have similar overall HRQoL compared to the general population, surgery has an impact on return to work and cognitive function.

In the last few decades, there has been an increase in the incidence of meningiomas being diagnosed among asymptomatic individuals, attributed to easier access to advanced neuroimaging techniques.^1,2^ Epidemiological surveys have shown that in current practice, approximately 40% of all diagnosed meningiomas are incidental.^3,4^ Management decisions can be made based on radiological features of the tumor and clinical characteristics of the patient. The European Association of Neuro-Oncology guidelines from 2021 suggest that “watch and scan” is the appropriate course of action in most asymptomatic patients.^5^ In individuals with radiological progression, or strong personal preference, surgery could be offered to asymptomatic patients. However, in such cases, there is no clear consensus on whether to offer surgery or not, as the risks of complications may outweigh the benefits.^6–8^

Brain surgery can have a major impact on health-related quality of life (HRQoL).^9^ HRQoL is a multidimensional concept encompassing commonly valued aspects of life and their impact on one’s overall sense of well-being and health functioning.^10^ In current literature, the effects of symptomatic meningiomas on HRQoL are reported, but data on the effects of surgery in asymptomatic meningioma patients is scarce. Both the conservative “watch and scan” as well as surgical intervention have been associated with decrease in quality of life (QoL), further complication decision-making in the patient group.^11,12^ In this population-based study of surgically treated asymptomatic meningiomas, we used patient-centered report forms from Stockholm County (Sweden) to evaluate the clinical course and their long-term HRQoL. Since patients were considered asymptomatic at the time of surgery, we compared their long-time post-surgery HRQoL with normative data from a matched subset of the general population, thereby broadening the understanding of HRQoL in this specific patient group.

## Hypothesis

Asymptomatic meningioma patients undergoing surgical resection have equivalent health-related quality of life at long-term follow-up after surgery compared to a matched subset of the general population.

## Materials and Methods

### Study Population

**Figure 1.**
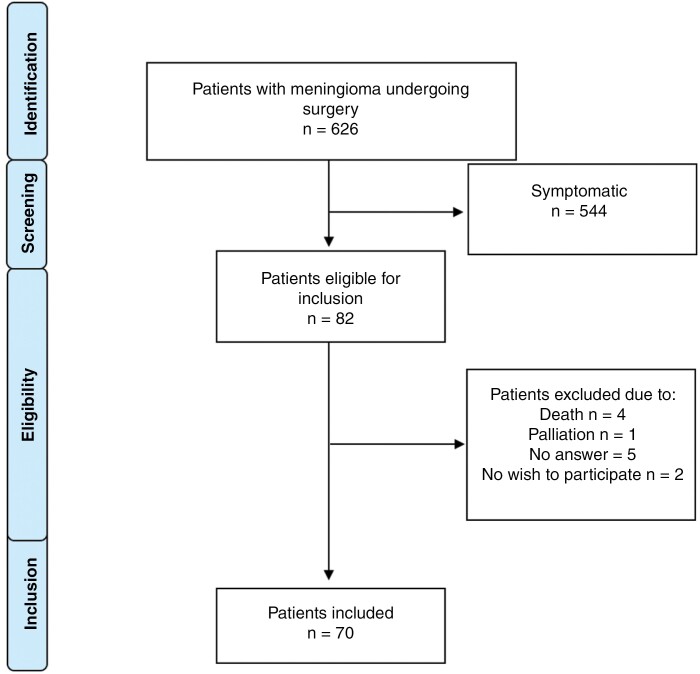
Flowchart of search strategy.

Patient data were retrospectively collected from a consecutive cohort of adult (≥18 years) meningioma patients treated between January 1, 2007, and June 30, 2013 at Karolinska University Hospital.^13^ In this cohort of 626 patients, 82 patients were considered asymptomatic. To validate that the study population were asymptomatic, the Swedish Brain Tumor Registry was utilized. After the verification of patients being asymptomatic at the time of surgery in both the research database and the quality registry Swedish Brain Tumor Registry, patients were contacted by mail in 2017. The mail consisted of study information, the questionnaire, and a written consent form. Patients who returned the written informed consent form and questionnaire were included in the study (Figure 1). This work was performed in line with the principles of the Declaration of Helsinki. This study was approved by the Swedish Regional Ethics Committee under DNR 2018/16-31/2.

### Questionnaire

The questionnaire administered to the asymptomatic meningioma study participants was based on the Stockholm Public Health survey (see below). This questionnaire was constructed by choosing a subset of questions from the survey deemed interesting to our study population, with the aim to map the patient’s life situation including HRQoL, lifestyle, socioeconomic factors, and familial factors. A translated version of the patient questionnaire is provided in the Supplementary Materials. The EuroQoL-5 dimension, 3 levels of response (EQ-5D 3L) questionnaire is a part of the survey, and this questionnaire is a generic and preference-weighted measure of HRQoL.^14^ It has previously been applied to a wide range of health conditions and treatments as well as population-based health surveys and is therefore suitable for comparison with the general population.^15,16^ The results of EQ-5D 3L questionnaire were transferred to a utility index ranging from −0.594 to 1, where higher scores indicated higher quality of life.^17^ Value sets were calculated in accordance with Burström et al.^18^ The visual analog scale is an instrument as part of the EQ-5D 3L which measures individuals perceived health in relation to the highest imaginable health. The scale reaches from 0 to 100, where 100 is the best imaginable health status. To intercept more disease-specific complications, certain questions were asked specifically to the patients. These were questions regarding possible postoperative symptoms such as epilepsy and neurological deficits, and medications used. Patients were asked to report their occupation both before and after surgery, as well as whether they returned to work postoperatively, and if so, the duration of time before returning.

### Normative Data From General Population

Normative data for EQ-5D 3L and data regarding general health, lifestyle, socioeconomic, and familial factors in an age and sex-matched control group were taken from the Stockholm Public Health Survey 2010, with data extracted from variables matching the questions asked to the asymptomatic meningioma patients. Due to the COVID-19 pandemic, there was a delay until 2022 in the acquisition of data from the general population. The Stockholm Public Health Cohort is a prospective, population-based cohort mapping the determinants and consequences of significant contributors to the current burden of disease in Stockholm County.^19^ The cohort involves area-stratified, cross-sectional random samples of the population of Stockholm County aged ≥ 18 years. To date, the cohort includes almost 150 000 participants. Matching with the meningioma patients was undertaken using propensity score, applying a nearest neighbor criteria, based on age and gender in the case cohort and general population. The estimation of the propensity score was made using the R package, MatchIt (version 4.5.4).

### Clinical Characteristics

The clinical data extracted from patient medical records included age at surgery and sex, previous comorbidities, Karnofsky Performance Score,^20^ lengths of surgery, World Health Organization (WHO) grade (as classified at time of surgery),^21,22^ Simpson grade,^23^ tumor location, postoperative complications according to Landriel-Ibanez,^24^ course of disease and adjuvant therapy, date of last radiological follow-up, and death before end of follow-up June 1, 2022.

Recurrence was defined as the presence of a new lesion on a subsequent radiological examination in a patient who had previously undergone radical removal, as determined by Simpson grading, and confirmed on postoperative brain scanning. The location of the tumor was classified as falx and parasagittal; sphenoid wing; convexity; olfactory, planum and sella; tentorium; petroclival and brain stem; intraventricular; and other infratentorial.

### Statistics

Statistical analyses were performed using IBM SPSS version 29 software. Normality for continuous variables were calculated using Kolmogorov-Smirnov test. All tests were 2-sided, and for descriptive statistics central tendencies were calculated as percentages, means with standard deviation (SD), or medians with first and third quartiles as appropriate. A *P* value of < .05 was considered statistically significant. Continuous data was analyzed using independent sample *t*-test or Mann–Whitney U test as appropriate. Similarly, categorical variables were analyzed using Pearson’s Chi-square or Fishers exact test.

## Results

### Patient Selection

The patient selection process is outlined in Figure 1. A total of 82 patients with surgically removed incidental and asymptomatic meningiomas were identified. Four deceased patients and one patient under palliative care for an unrelated cause were excluded from the study. Five patients never responded, and 2 patients declined to participate. Thus, 70 patients were included in the study, hence with an inclusion rate of 91%. For responders, the mean time from surgery to answering the questionnaire in 2017 was 6.7 years (SD 1.8 years).

A corresponding number of controls were selected according to the above-mentioned matching process. Mean age as of 2017 was 63 years among both patients and controls. In both groups, 67% of included individuals were of female sex.

### Baseline Characteristics

For tumor and surgical outcome characteristics of patients with surgically removed asymptomatic meningioma, see Table 1. At the time of surgery, the mean age was 55 years and all but one had a Karnofsky Performance Score above 70 preoperatively. The convexity was the most common tumor location (33%) followed by the falx and parasagittal (21%) and sphenoid wing (17%). Laterality was distributed equally (50% on each side), and most meningiomas were solitary (96%).

**Table 1:** Individual and Tumor Characteristics Among Patients

Variables	*n* (%)
Age at surgery, mean (SD)	55 (11)
KPS > 70	69 (99)
Tumor location	
Convexity	23 (33)
Falx and parasagittal	15 (21)
Tentorium	3 (4)
Sphenoid wing	12 (17)
Petroclival/brain stem	4 (6)
Olfactory/planum/sella	10 (14)
Other	3 (4)
Perifocal edema	13 (19)
Mainly left-sided tumor	35 (50)
Solitary meningioma	67 (96)

Most tumors were removed with gross total resection, ie, Simpson grade I and II (54 and 27% respectively). All but one case had a WHO grade 1 tumor as graded by the 2007 WHO classification.^22^ Twenty-nine percent of the cases had a complication as classified by Landriel-Ibanez, with the most common complication being a non-life threatening deviation from postoperative course requiring drug administration (17%, grade 2b). At the end of follow-up, 6% of cases had been diagnosed with recurrence, and 3 % with progression of a known remnant. A total of 12 patients (17%) received some kind of treatment after primary surgery, either another surgery due to the progress of remnant or recurrence, or stereotactic radiosurgery due to subtotal resection, progress of remnant or recurrence. At the end follow-up period, 2.9% of the patients had died (Table 2).

**Table 2.** Outcome After Surgery Among Patients

Simpson grade	*n* (%)
I	38 (54)
II	19 (27)
III	0 (0)
IV	12 (17)
Missing	1 (1)
WHO grade^*^	
1	69 (99)
2	1 (1)
3	0 (0)
Postoperative complication within 30 days (Ibanez)	
1a	1 (1)
1b	12 (17)
2a	1 (1)
2b	4 (6)
3a	0 (0)
3b	2 (3)
Course of disease	
No recurrence	53 (76)
Recurrence	4 (6)
Stable disease	10 (14)
Progress of remnant	2 (3)
Missing	1 (1)
Adjuvant treatment due to subtotal resection/recurrence/progress of remnant	
Reoperation	3 (4)
Radiotherapy	9 (13)
Death before end of follow-up (01/06/2022)	2 (3)

^*^As graded at time of surgery.

Table 3 summarizes lifestyle and occupational data for both patients and controls. Before surgery, 86% of patients were employed, while 6% were retired and 4 % were on long-term sick leave. After surgery, only 60% of patients returned to their previous occupation. Among those who did return to their previous occupation, excluding those already retired, half of patients did so within 3 months after surgery, and an additional 26% returned after 3–6 months of time. 8% of cases returned after 6–12 months, and 16% after more than 12 months. The primary reason for not returning to a previous occupation after surgery was retirement, which was stated as the reason for no return to work by half of all patients. The second most common reason not to return to previous occupation was due to symptoms related to stress, fatigue, concentration issues, and difficulty handling the pressure to perform at the workplace, which 28% of patients stated.

**Table 3.** Lifestyle and Occupancy of Patients and Controls

Lifestyle and working conditions	Patients, *n* (%)	Controls, *n* (%)	*P*-value
**Preoperative occupation**		N/A	
Work	60 (86)		
Unemployed	1 (1)		
Long-term sick leave	3 (4)		
Retired	4 (6)		
Missing	2 (3)		
Did you return to your occupation after surgery?		N/A	
Yes	42 (60)		
No	25 (36)		
Missing	3 (4)		
If yes, after how much time? ^*^		N/A	
Within 3 months	19 (50)		
3–6 months	10 (26)		
6–12 months	3 (8)		
> 12 months	6 (16)		
If no, why?		N/A	
Retirement	12 (48)		
Stress, pressure to perform, fatigue, issues concentrating, etc.	7 (28)		
Hemiparesis	1 (4)		
Loss of olfactory sense	1 (4)		
Chronic pain	1 (4)		
Missing	3 (12)		
How many hours of work per week			.29
More than 45 hours	4 (10)	7 (19)	
36–45 hours	18 (46)	22 (60)	
20–35 hours	7 (18)	6 (16)	
1–19 hours	2 (5)	2 (5)	
Missing	8 (21)	0 (0)	
Sick leave past 12 months^*^			*.04*
None	8 (21)	15 (41)	
Yes, one time	10 (26)	12 (32)	
Yes, 2–4 times	11 (28)	6 (16)	
Yes, 5–9 times	2 (5)	2 (5)	
Yes, 10 or more times	1 (3)	0 (0)	
Missing	2 (5)	0 (0)	
Total number of sick leave days past 12 months			*.01*
1–7 days	12 (46)	18 (75)	
8–30 days	9 (35)	5 (21)	
31–90 days	2 (8)	1 (4)	
More than 90 days	2 (8)	0 (0)	
Missing	1 (4)	0 (0)	
Smoking	6 (9)	18 (26)	*<.001*
Snuff	2 (3)	8 (11)	*<.001*
Cohabitation	44 (63)	57 (81)	*.01*
If yes: ^**^			
Partner	41 (93)	48 (84)	
Parents	1 (2)	0 (0)	
Other adult	0 (0)	2 (4)	
Children	12 (27)	21 (37)	
Caring for family member	11 (16)	8 (11)	.66
How many hours, median (Q1-Q3)	10 (1–30)	3.5 (2–13)	.6
Do you have someone who can give you support?			*.04*
Yes, always	32 (46)	47 (67)	
Yes, most of the time	22 (31)	17 (24)	
No, rarely	11 (16)	3 (4)	
No, never	5 (7)	2 (3)	
Have you in the past 12 months participated in any regular activities	34 (49)	42 (60)	.15

^*^If retired at time of surgery, not included.

^**^Total may exceed the number of patients with cohabitation as you can live with different types of individuals.

There was no difference in the number of hours working per week between patients and controls (*P* = .29). Working 36–45 hours per week was reported as the most common (46, respectively, 60%), followed by 20–35 hours (18, respectively, 16%) and more than 45 hours (10, respectively, 19%). 20% of the participants did not report the number of hours worked per week. The occurrence of sick leave and the total number of days of sick leave were significantly different between the groups (*P* = .04 and *P* = .01, respectively). Among the patients 20% had not been on sick leave for the past 12 months, 26% had been on sick leave once in the last year, and 28% 2 to 4 times the past year. Corresponding figures for the controls were 40%, 32%, and 16%, respectively. In terms of the total number of days on sick leave, there was a significant difference between the 2 groups, with patients having more days of sick leave than controls (*P* = .04).

It was significantly more common for the controls compared to the patients to smoke and consume snuff tobacco (26% vs. 9%, *P* < .001; 11% vs. 3%, *P* < .001). There was also a difference in cohabitation between the groups. Significantly more of the controls cohabited with either a partner, parent, child, or some other adult (81% vs. 63%, *P* = .013). The groups showed no significant difference in the occurrence of caring for a family member, nor the median time each week spent caring for a family member. There was a significant difference between the patients and controls in terms of the supportive system, where 67% of the controls felt they always had someone they could turn to if needed of support, with a corresponding 46% of the patients.

Patients and controls did not significantly differ in the prevalence of comorbidities, except for diabetes and angina pectoris where the occurrence of disease was higher among patients (Table 4). After meningioma surgery, 17% of the patients reported double vision, 36% speech difficulties, 14% half-sided weakness, and 6% epileptic seizures. Additionally, 11% of patients reported use of medication with antiepileptic effect. At long-term follow-up 47% of patients and 31 % of the controls stated that they had some kind of serious illness, ailment after accident, handicap, or long-term health problem (*P* = .06). However, among the patients who did state some kind of long-term health issue, significantly more individuals among the patients felt that their health issue hindered them in their everyday life to some or a high degree (88% vs. 59%, *P* = .03). Interestingly, there was no difference between groups in persistent fatigue. However, it was significantly more frequent for patients to suffer from difficulty sleeping (*P* = .02) and sound sensitivity (*P* < .001, Supplementary Table 1).

**Table 4.** Self-Perceived Health of Patients and Controls

Perceived health and symptoms	Patients, *n* (%)	Controls, *n* (%)	*P*-value
Self-reported diagnoses			
Diabetes	10 (14)	1 (1)	*.003*
COPD	1 (1)	1 (1)	.13
Psoriasis	1 (1)	1 (1)	.13
Hyperlipidemia	15 (21)	7 (10)	.06
Angina pectoris	2 (3)	0 (0)	*.04*
Heart failure	1 (1)	0 (0)	.36
Depression	12 (17)	11 (16)	.07
How would you assess your general state of health?			.3
Very good	15 (21)	16 (23)	
Good	29 (41)	37 (53)	
Moderate	21 (30)	11 (16)	
Bad	4 (6)	4 (6)	
Missing	1 (1)	2 (3)	
Do you have a serious illness, ailment after accident, handicap, or long-term health issue			.06
Yes	33 (47)	22 (31)	
No	35 (50)	46 (66)	
Missing	2 (3)	2 (3)	
If yes, does it reduce your ability to work or otherwise hinder you in your everyday life?			*.03*
Yes, to high degree	12 (36)	5 (23)	
Yes, to some degree	17 (52)	8 (36)	
No	4 (12)	9 (41)	

**Figure 2.**
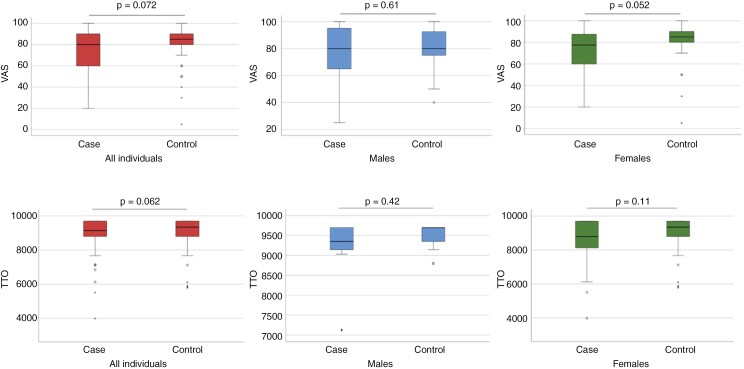
EQ-5D-3L visual analogue scale and TTO value comparison of patient versus control between all individuals, females, and males.

When analyzing EQ-5D 3L index value and EQ-5D visual analog scale there were no significant differences either when analyzing all individuals (*P* = .072), males (*P* = .61), or females (.052; Figure 2). In Supplementary Table 2 the resulting TTO values in different stratified age groups are shown, with no significant difference between patients and controls in any of the age categories.

## Discussion

In this population-based matched cohort study, involving 70 asymptomatic meningioma patients who underwent surgical treatment, we found no difference in HRQoL between the patients and their matched controls from the general population. No difference could be seen either when analyzing as a whole group, or when analyzing subgroups of the different sexes, or different age groups. This was in line with the hypothesis that these patients have an equivalent HRQoL compared to the general population, implying that meningioma surgery for asymptomatic patients does not severely reduce long-term quality of life (QoL) in this patient group.

In this study, both groups had similar rates of comorbidities, except for diabetes and angina pectoris, which were more prevalent among patients. Despite this, self-assessment of general health did not significantly differ between the 2 groups, with the majority of both patients and controls rating their health as good or very good. There is a possibility that the effects of surgery on QoL have been diluted a mean of 6.7 years after surgery, with the idea that patients with time could accommodate postoperative lowering of HRQoL.^25^ There are few previous studies on HRQoL in surgically treated incidental meningioma, but a previous study using the Short form 36 health survey (SF-36) to assess QoL in radiologically suspected incidental meningioma, found that patients reported lower scores in the domains vitality and general health.^26^ SF-36 was also used in a study evaluating HRQoL in actively monitored and surgically removed meningioma, which found compared to a normative population clinically relevant and significantly worse HRQoL scores in the domains of physical and emotional health problems, energy/fatigue as well as social functioning.^27^ In a surgical series of symptomatic meningioma, 20% reported worsening of patient-reported QoL after meningioma treatment.^28^ When studying surgically removed spinal meningioma, a similar pattern as in our study was seen, with no difference in HRQoL as measured by EQ-5D-3L between patients and the general public.^29^

Patients with meningioma have impaired HRQoL both before and years after surgery, as shown by a recent systematic review.^30^ In cases of asymptomatic meningioma patients experiencing a high degree of distress, it may be tempting to offer surgery in order to reduce the patients’ worry, in an attempt to improve QoL. However, distress and QoL have been shown not to change in asymptomatic patients who receive surgical treatment, compared to conservatively treated patients, and the surgically treated patients had equally high levels of distress and reduction in QoL.^12^ Collectively the above-mentioned problems may affect the patient’s ability to work and engage in social life and should be considered and discussed with the patients if treatment for an incidental meningioma is considered.

Treatment, tumor size, extent of surgical resection, and histologic grade of the tumor are all factors that influence the impact of a meningioma on a patient’s HRQoL.^31,32^ A history of depression or sick leave may also independently affect the response to a meningioma diagnosis and meningioma treatment.^33^ While previous studies have shown that treating symptomatic meningiomas is associated with an increase in HRQoL, it is prudent to be restrictive in offering treatment for asymptomatic meningiomas.^34^ Particularly caution is advised among elderly patients as surgery in this group is associated with an elevated risk for complications.^35^ The majority of asymptomatic meningiomas do not grow during observation over 5 years, and only a small percentage become symptomatic, further underpinning the importance of avoiding surgical treatment when possible.^36^

Among patients in this study, only 60% returned to their preoperative occupation following surgery, with many choosing to retire. The reasons for this choice were not fully explored. Cognitive issues were another common reason for not returning to previous occupation, affecting more than a quarter of patients. These issues included lowered stress tolerance, fatigue, concentration issues, and difficulties handling pressure to perform in the workplace. A previous study has shown that among actively monitored and surgically removed meningioma, patients in employment had better EORTC Core Quality of Life of Cancer Patients questionnaire (QLQ-C30) score.^27^ Just as in this study it is difficult to know whether employment leads to better HRQoL, or if better HRQoL helps patients stay in employment. Among those who did return to work, almost 16% did so after more than a year of sick leave. In line with this, a recent Swedish study reported that over 40% of meningioma patients of working age were still on sick leave 2 years after surgery.^33^ This underscores the impact of meningioma surgery on the ability to maintain presurgical levels of occupation, which is also true for asymptomatic patients. As stated above, this could be attributed to postoperative fatigue, with more than 40% of patients reporting emotional and cognitive difficulties following meningioma surgery.^37–39^

Many cognitive factors contribute to the ability to function in the workplace, and in a previous study of radiologically suspected meningiomas, patients performed worse compared to their healthy controls on working memory and motor speed.^26^ This finding was however opposed in another study.^40^ When asked specifically about persistent fatigue the results of this current study were similar between patients and controls. Fatigue is however already a frequent symptom in the general population, which might contribute to the similarities between our groups.^41–43^ Patients did however report a significantly higher frequency of both difficulty sleeping and sound sensitivity, which could potentially indicate lasting emotional and cognitive difficulties. In line with this, meningioma patients have also been shown to have a 50% increase in the use of both antidepressants and anxiolytics 2 years after surgery compared to 2 years prior to surgery.^44^

The experience of life being hindered by illness, handicap, or ailment extends beyond the workplace; as mentioned earlier, it can also significantly affect social life. In our study, patients reported significantly lower rates of cohabitation, and support systems for emotional aid than controls. The underlying reasons for this are not fully understood, yet it is plausible to speculate that factors such as the inability to continue working, early retirement, and cognitive difficulties could adversely affect personal relationships. Previous studies have also shown that meningioma are more common among nulliparous women.^45^ The above should be further explored to better understand if detachment occurs before or during disease trajectory in order to be able to give patients more comprehensive information regarding the effects of surgery, as well as sufficient support postoperatively to overcome such issues in the best way possible.

### Strengths and Limitations

A strength of the present study is the access to a relatively large cohort of consecutively treated asymptomatic meningioma patients, with highly detailed information on their HRQoL, and a matched control group from the general population with the same data for comparison. The questionnaire used for patients has been based on a comprehensive public health survey conducted by the Stockholm Region, consisting of validated questions, and spanning a multitude of areas important for QoL and overall health. The patient questionnaire response rate was 91%, reducing the risk of selection/response bias. However, the questionnaire used has not been specifically validated for meningioma patients, and as a result, there may be specific issues relating to having meningioma that remain unexplored. EQ-5D is however a generic questionnaire designed and validated to be applicable to many areas of medicine.

Although in this context, an even larger cohort of patients could have revealed other findings, another limitation to the study is the time between surgery and answering the questionnaire, which could span anywhere from 4 to 10 years. Since surgical removal of asymptomatic meningiomas is not so common in our part of the world, a formal power analysis was not performed, and a longer retrospective inclusion period was opted for to maximize cohort size. It was thereto deemed important with proper matched controls from the same environment, hence using the SPHC was considered crucial, why the inclusion of cases was not expanded to other areas of Sweden.

Over time, issues related to HRQoL may change for better or worse. This was a cross-sectional study, where those with shortest interval from surgery could theoretically still be in a postoperative improvement phase, and some individuals with longer follow-up times may have deteriorated over time to lower health states due to the natural consequences of aging. However, previous studies have shown that improvements in HRQoL are expected to be steepest in the first year post-surgery.^33^ Matching was made between cases and controls from the general public based on age, which should account for some of the deterioration in HRQoL due to aging. Future longitudinal studies on both short-term and long-term HRQoL among asymptomatic meningioma patients are encouraged. Also, more detailed matching criteria, such as level of education or neighborhood of residence, could perhaps influence future results, as these factors are known to significantly influence QoL.

### Summary and Future Directions

In conclusion, this study found no significant difference in HRQoL as measured by EQ-5D-3L between patients and their matched controls from the general population. However, among those with health issues, symptoms, or ailments, patients stated a greater impact on work- and everyday life, and a significant proportion of patients did not return to their previous occupation after surgery. Many chose to retire, and a quarter of patients stated cognitive and emotional symptoms as their cause of altered occupancy. Detachment from social life was also significantly more common among patients, with lower levels of cohabitation and smaller emotional support systems. The effects of the above-mentioned should be thoroughly discussed before making the decision on treatment of asymptomatic meningioma, and further studies in larger cohorts are encouraged to expand the best of our knowledge on this topic.

## Supplementary material

Supplementary material is available online at *Neuro-Oncology* (https://academic.oup.com/neuro-oncology).

npae047_suppl_Supplementary_Tables

## Data Availability

Data are available upon contact with the corresponding author.
