# Supplementary material for: Health-related quality of life in surgically treated asymptomatic meningioma patients: A population-based matched cohort study
Source: Neurooncol Pract. 2024 Nov 15;11(6):723–32. doi: 10.1093/nop/npae047 (PMC11567747; doi:10.1093/nop/npae047)
Supplement: npae047_suppl_Supplementary_Tables [file npae047_suppl_supplementary_tables.docx]

**Supplementary table 1: Self-perceived symptoms of patients and controls**

| **Symptoms** | **Patients, n (%)** | **Controls, n (%)** | **p-value** |
| --- | --- | --- | --- |
| **Headache/migraine** |  |  | 0.14 |
| No | 38 (54) | 48 (69) |  |
| Yes, slight bother | 26 (37) | 16 (23) |  |
| Yes, great bother | 6 (9) | 2 (3) |  |
| Missing | 0 (0) | 4 (6) |  |
| **Persistent fatigue** |  |  | 0.22 |
| No | 35 (50) | 43 (61) |  |
| Yes, slight bother | 23 (33) | 18 (26) |  |
| Yes, great bother | 11 (16) | 5 (7) |  |
| Missing | 1 (1) | 4 (6) |  |
| **Difficulty sleeping** |  |  | *0.02* |
| No | 32 (46) | 46 (66) |  |
| Yes, slight bother | 27 (39) | 18 (26) |  |
| Yes, great bother | 10 (14) | 4 (6) |  |
| Missing | 1 (1) | 2 (3) |  |
| **Anxiety or worry** |  |  | 0.14 |
| No | 38 (54) | 47 (67) |  |
| Yes, slight bother | 27 (39) | 19 (27) |  |
| Yes, great bother | 3 (4) | 2 (3) |  |
| Missing | 2 (3) | 2 (3) |  |
| **Snoring** |  |  | 0.71 |
| No | 42 (60.0) | 41 (59) |  |
| Yes, slight bother | 23 (33) | 21 (30,0) |  |
| Yes, great bother | 3 (4) | 3 (4) |  |
| Missing | 2 (3) | 5 (7) |  |
| **Tinnitus** |  |  | 0.58 |
| No | 43 (61) | 46 (66) |  |
| Yes, slight bother | 20 (29) | 19 (27) |  |
| Yes, great bother | 6 (9) | 2 (3) |  |
| Missing | 1 (1) | 3 (4) |  |
| **Sound sensitivity** |  |  |  |
| No | 34 (49) | 57 (81) | *<0.001* |
| Yes, slight bother | 27 (39) | 9 (13) |  |
| Yes, great bother | 8 (11) | 1 (1) |  |
| Missing | 1 (1) | 3 (4) |  |
| **Double vision** |  | N/A |  |
| No | 56 (80.0) |  |  |
| Yes, slight bother | 10 (14) |  |  |
| Yes, great bother | 2 (3) |  |  |
| Missing | 2 (3) |  |  |
| **Speech difficulties** |  | N/A |  |
| No | 43 (61) |  |  |
| Yes, slight bother | 22 (31) |  |  |
| Yes, great bother | 3 (4) |  |  |
| Missing | 2 (3) |  |  |
| **Half-sided weakness** |  | N/A |  |
| No | 58 (83) |  |  |
| Yes, slight bother | 6 (9) |  |  |
| Yes, great bother | 4 (6) |  |  |
| Missing | 2 (3) |  |  |
| **Epileptic seizures** |  | N/A |  |
| No | 65 (93) |  |  |
| Yes, slight bother | 2 (3) |  |  |
| Yes, great bother | 2 (3) |  |  |
| Missing | 1 (1) |  |  |
| **Postoperative use of AEDs** | 8 (11) | N/A |  |

**Supplementary table 2: TTO values in different age groups**

| **Age group** | **Patient** | **Control** | **p-value** |
| --- | --- | --- | --- |
| 30-39, median (Q1-Q3) | 0.925 (0.880-0.969) | 0.735 (0.590-0.880) | 0.22 |
| 40-49, median (Q1-Q3) | 0.914 (0.745-0.969) | 0.969 (0.947-0.969) | 0.14 |
| 50-59, median (Q1-Q3) | 0.935 (0.880-0.969) | 0.935 (0.880-0.969) | 0.89 |
| 60-69, median (Q1-Q3) | 0.880 (0.767-0.969) | 0.935 (0.880-0.944) | 0.06 |
| 70-79, median (Q1-Q3) | 0.935 (0.880-0.969) | 0.952 (0.921-0.969) | 0.24 |
| 80-89, median (Q1-Q3) | 0.925 (0.914-0.935) | 0.914 (0.909-0.942) | 0.77 |
